# Fish Hacks: Hybridizing Stand-Alone Zebrafish System Plumbing and Pumps to Extend and Improve Function

**DOI:** 10.1089/zeb.2023.0011

**Published:** 2023-06-09

**Authors:** Jacob Starkey, Eric J. Horstick

**Affiliations:** ^1^ Department of Biology, West Virginia University, Morgantown, West Virginia, USA.; ^2^ Department of Neuroscience, West Virginia University, Morgantown, West Virginia, USA.

**Keywords:** DIY, water pump, system maintenance, stand-alone system, aquatics, zebrafish

## Abstract

One of the greatest expenses in running a zebrafish laboratory is the aquatic systems used for housing. These critical pieces of equipment are essential and incorporate components undergoing constant activity in pumping water, monitoring, dosing, and filtration. The systems available on the market are robust, yet ongoing activity eventually leads to the need for repair or replacement. Moreover, some systems are no longer commercially available, impairing the ability to service this critical infrastructure. In this study, we demonstrate a do it yourself (DIY) method to re-engineer an aquatic system's pumps and plumbing, which hybridizes a system no longer commercially available with components used by active vendors. This transition from a two external pump Aquatic Habitat/Pentair design to an individual submerged pump Aquaneering-like plan saves funds by expanding infrastructure longevity. Our hybridized configuration has been in uninterrupted use for >3 years, supporting zebrafish health and high fecundity.

Zebrafish have fast emerged as a powerful model for understanding development and behavior.^1,2^ The expanding use of zebrafish in research has come with an increasing need to ensure that the care of these fish is consistent and optimized.^3,4^ Maintaining the operation of aquatic systems is central to proper zebrafish care, which improves health and fecundity.^5–7^ Although some institutions can support large decentralized aquatic systems, many utilize stand-alone rack systems. Stand-alone systems have been in use for decades in the fish community. Similarly, designs for entirely home-made do it yourself (DIY) systems of different scales are increasingly being used.^8–12^

However, in many cases, such stand-alone or home-made systems, unlike decentralized facilities, the care and upkeep can fall onto an individual laboratory. Maintenance varies from frequently replacing filters and probes to the costlier replacement of pumps and electronics. Depending on available in-house or commercial expertise, the required time and cost for these repairs can vary greatly. Therefore, having plans to make effective changes to a system saves time and grant dollars by extending the utility and longevity of their aquatic system.^8,13^ In this study, we outline a DIY approach to replacing water pumps and associated plumbing to efficiently extend an aquatic system's lifespan.

The original pump design for Aquatic Habitat/Pentair systems involved a single water intake that distributed water to dual external water pumps. A three-way valve pumped water to filtration and the tanks (Fig. 1A). In our laboratory, we inherited one of these systems, yet the pumps were beyond recommended usage resulting in excessive noise and vibration through the housing tanks. In this system design, vibrations spread to the zebrafish tanks since the pumps are bolted to a rigid base that is integrated into the rack structure. To improve the long-term pump function and ease of use for this system, we remodeled the water flow system.

**FIG. 1.**
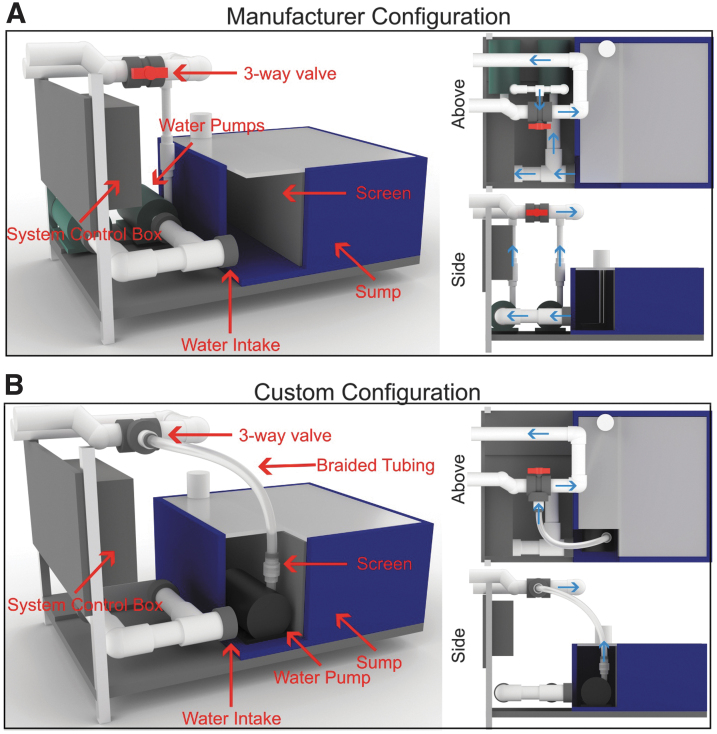
Modification of zebrafish housing system's plumbing and pumps. **(A)** Manufacturer configuration of an Aquatic Habitat/Pentair housing system. This system originally utilized two external water pumps to circulate water from the sump to the tanks. **(B)** The custom pump and plumbing configuration show the changes that allow for the use of a single submersible pump. *Blue arrows* indicate water flow direction from the sump to the system.

We re-engineered the water flow by removing the external pumps and switching to a single submerged pump, a Danner Supreme 2600 GPH (gallons per hour), placed within the sump, which we rationalized would be much easier to replace in the future (Fig. 1B). In addition, a submerged pump ideally would transmit less vibration through the system, even if failing, by not being directly bolted to the base structures. For detailed materials and methods on this remodeling, see Supplementary Data S1, Supplementary Table S1, and Supplementary Figures S1–S3. For the past 3 years of constant use after this modification, there have been no observed impacts on water pressure or circulation throughout the housing racks. The only additional maintenance is the periodic replacement of the extra water flow tubing used to integrate the submerged pump into the rebuilt plumbing.

In summary, we re-engineered the water flow of an older model stand-alone fish system by hybridizing existing and new components. Our design is straightforward and easily applied with minimal tools and time. Overall, our methods can extend the viability of systems with limited available commercial support. Although in this study we provide precise instructions for one particular system to system modification, we believe the concepts can be generalized for other stand-alone or home-made systems. For instance, any pump with flow rates capable of reaching the vertical heights of the system in question should be compatible (typical metric provided for pumps). In addition, a broad spectrum of polyvinyl chloride (PVC) fittings, adapters, and connectors are available to establish flow paths that can accommodate and integrate a different water pump, regardless of starting platform and may just require trial and error testing.

Important considerations for attempting modifications on different systems than what is described in this study are space and current system assembly (e.g., would a different pump fit and/or are all pieces glued and require cutting to remove). Nonetheless, the availability of diverse pumps and endless PVC components should make most systems adaptable. DIY modification to existing aquatic systems is a time and cost-effective strategy to maintain research activities and efficiently use grant dollars. This DIY example shows that crucial infrastructure in zebrafish laboratories can be easily adjusted to maintain operable life. Moreover, we consider this pump hybridization design a single example, and continued DIY efforts could propose additional modifications to improve function across more system styles or operations.

## Supplementary Material

Supplemental data
