# Supplementary material for: Fish Hacks: Hybridizing Stand-Alone Zebrafish System Plumbing and Pumps to Extend and Improve Function
Source: Zebrafish. 2023 Jun 9;20(3):122–5. doi: 10.1089/zeb.2023.0011 (PMC10280213; doi:10.1089/zeb.2023.0011)
Supplement: Supplemental data [file Suppl_Data.docx]

Supplementary Material

**Tools:**

Wrench/Socket wrench

Wire cutter

Rubber mallet

Flathead screwdriver

Tubing cutter

Plumber’s tape (any brand or variety is fine)

Plumbers wrench (optional)


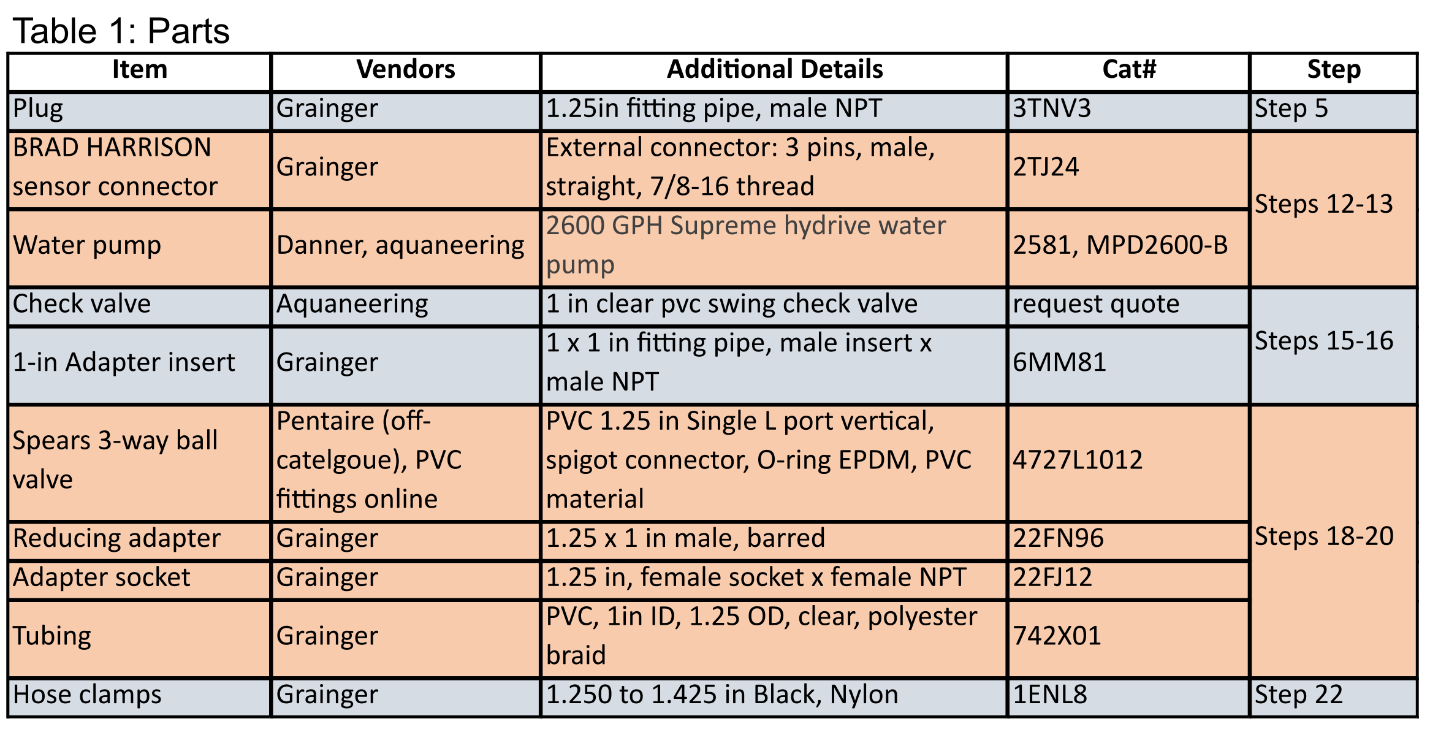


**Methods:**

1. Before starting, clean all components that will be in contact with system water. Any fish room standard cleaner is acceptable. Allow components to dry.

2. Turn off the system and allow time for water to drain out of lines into the sump

3. Use the drain valve (adjacent to 50 µm filter) to drain additional water out of the lines

4. Close UV bypass valve, UV inlet valve, and both water pump inlet valves to minimize water movement

5. From inside the sump, screw the PVC plug (see materials) into the water pump intake opening. Make sure to wrap plug threading with plumbers’ tape. The plug does not need to be threaded entirely in, just enough to block water flow

6. Now, you will begin disassembling the current plumbing for the external pumps. Around the pumps, identify the two pump inlet valves (before pumps) and two check valves (immediately above pumps, clear with internal flap to control water flow direction). All these pieces are connected to the current plumbing with union fittings (threaded screw connections) (**Figure 1**). Slowly loosen these four connectors and allow time for the water to drain. Once leaks stop, valves can be completely loosened and removed. Water pump intake line (from sump) can be left. Note: Loosen any stubborn connections using a plumber’s wrench.


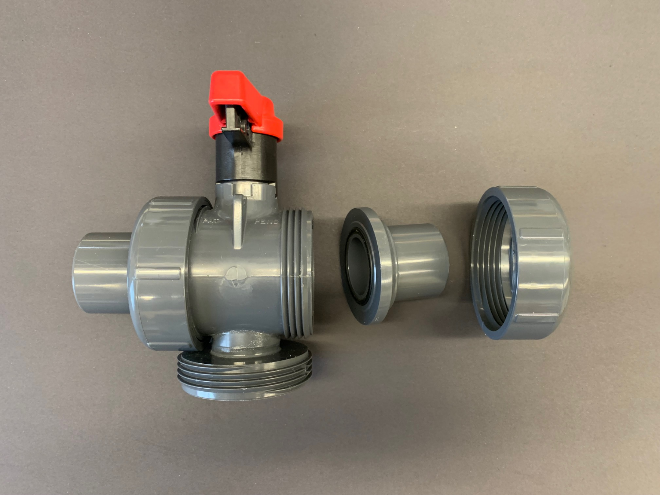


**Figure 1. 3-way valve with union fittings.** 3-way valve with an example assembled (left) and disassembled (right) union fitting.

7. Disconnect both pumps from the system control box.

8. Optional: The external pumps can be removed. Note: multiple wrenches or wrench and sockets will be needed to loosen pumps as they are secured with bolts through the entire base and secured with a hex nut underneath

9. Note the orientation of the water pump outlet 3-way valve (directs water flow to primary or filtration bypass plumbing path). The handle should be facing toward the system control box. Installation of the new 3-way valve will be reversed (handle toward the smaller heater control box)

10. Loosen all three union fittings on the 3-way valve and remove them. Cannot be salvaged as the original valve is likely glued to PVC piping

11. Replace with new 3-way valve in reverse (see step 7) with the handle toward the heater control box. Secure lateral union fittings with primary and bypass water flow routes. Leave the third union fitting assembled, which should be facing the direction of the system control box

12. Prepare Danner 2600 GPH pump to operate with Aquatic Habitats/Pentair system controller. Note: Do NOT plug in the pump or run when not submerged. Cut off the electrical plug and use a wire cutter to cut back approximately 1 inch of external wire covering. This will expose the 3 internal wires (white, black, green). Use the wire cutters to cut back approximately 0.25 in of covering from each wire to expose the internal copper wiring (**Figure 2A**).

13. Wire Danner 2600 GPH pump to BRAD HARRISON sensor connector. Disassemble the connector (2 threaded connection points). The connector should now be in three parts (end plug, body, and wire cap). Pass the end of the water pump electrical cord through the wire cap and body. Using a flathead screwdriver, loosen the set screws on the connector end plug so pump wires can be inserted. Note that the connector end plug has three positions labeled 1, 2, and 3. Insert the green, black, and white wires into positions 1, 2, and 3, respectively (**Figure 2B**). Tighten all three set screws and make sure each wire is securely anchored into the end plug. Secure the connector body with the end plug and last thread and tighten the wire cap onto the body (**Figure 2C-D**).

**Figure 2. Wiring pump to BRAD HARRISON connector. (A)** Pump wire covering cut exposing the three internal wires. **(B)** Wire inputs labeled 1,2, and 3 (left) and proper wire order (right) for the BRAD HARRISON connector. **(C)** BRAD HARRISON connector parts with attached wiring. **(D)** Assembled BRAD HARRISON connector (right) and plug prong view (right).


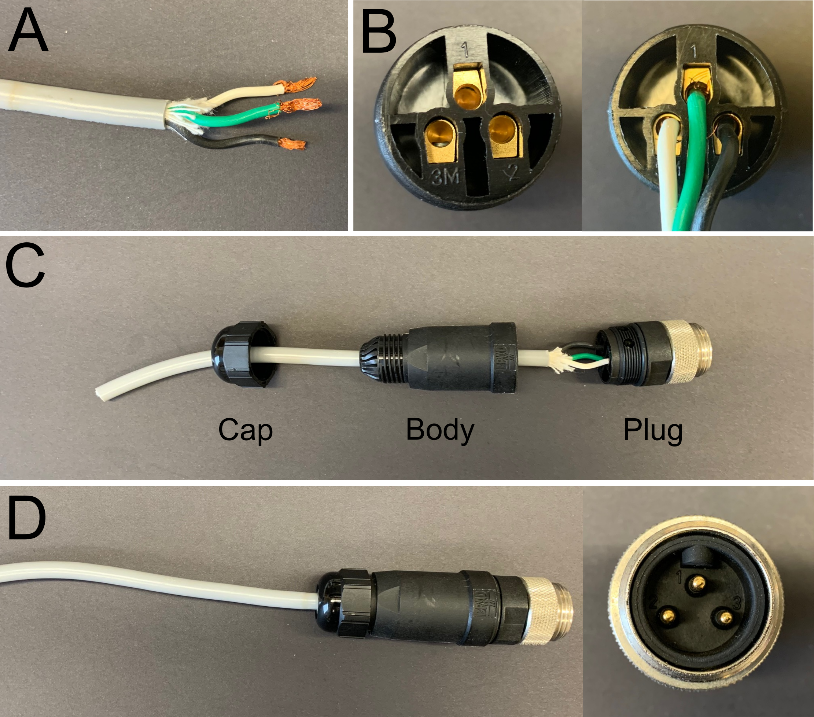


14. Submerge the pump into the sump adjacent to the water pump intake that was plugged earlier. The pump should be positioned between the water pump intake and biofilter media retaining wall. Place the intake screen onto the pump to prevent foreign objects from entering (screen provided with the pump). Avoid direct contact with the heating element. Note: we find it easier to position the pump in the sump before connecting other components.

15. Thread the check valve onto the outflow port of the pump (top opening). Use plumber’s tape for all threaded connections.

16. Thread the 1-in adapter insert (allows connection with tubing) onto the top of the check valve.

17. Connect the pump to the system control box. Either Pump 1 or Pump 2 port will work. It may be necessary to unplug surrounding connections to allow easier access to pump ports.

18. Attach the adapter socket to the open port of the 3-way valve. Keep the open port union fitting fully assembled. The smooth end of the adapter will slide onto the open port. Secure with several light taps with a rubber mallet. No PVC glue is necessary yet can be used if connections do not stay secure (**Figure 3A-B**).


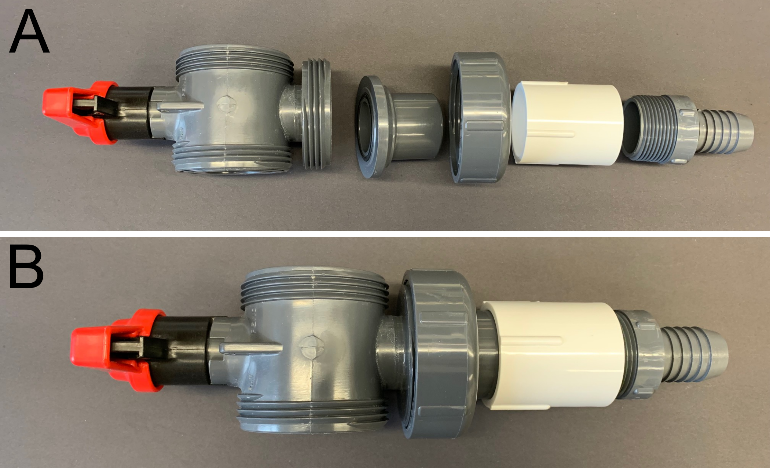


**Figure 3. 3-way valve to braided tubing connections. (A)** Left to right: 3-way valve with union fitting, adapter socket, and reducing adapter need to connect the braided tubing from pump to system. **(B)** 3-way valve after adapter assembly.

19. From the adapter socket secured to the 3-way valve, thread in the reducing adapter (**Figure 3A-B**).

20. Cut approximately 27in of tubing (braided) and connect to the open port of the 3-way valve and the pump (adapter above check valve). Adjust the length of the tubing as needed. Ensure tubing covers all of the ribbed area of the adapters at both the 3-way valve and pump connections. Secure tube/adapter connections using hose clamps by firmly squeezing the clasps of the clamp together.

21. Turn the system on and watch for leaks. If any leaks are observed, disassemble and reconnect as needed.

22. All tubing/adapter connections can be secured with hose clamps.
